# Dietary changes following a lifestyle-based intervention for dementia risk reduction – results from the AgeWell.de study

**DOI:** 10.1007/s00394-024-03563-z

**Published:** 2024-12-30

**Authors:** Andrea E. Zülke, Iris Blotenberg, Melanie Luppa, Margrit Löbner, Juliane Döhring, Martin Williamson, Robert P. Kosilek, Irina Michel, Anke Oey, Christian Brettschneider, Jochen Gensichen, David Czock, Birgitt Wiese, Hans-Helmut König, Thomas Frese, Hanna Kaduszkiewicz, Wolfgang Hoffmann, René Thyrian, Steffi G. Riedel-Heller

**Affiliations:** 1 Institute of Social Medicine, Occupational Health and Public Health (ISAP), University of Leipzig, Philipp Rosenthal Str. 55, 04103 Leipzig, Germany; 2 German Center for Neurodegenerative Diseases (DZNE), Site Rostock/Greifswald, Greifswald, Germany; 3 Institute of General Practice, University of Kiel, Kiel, Germany; 4 Institute of General Practice and Family Medicine, LMU University Hospital, LMU Munich, Munich, Germany; 5 State Health Department of Lower Saxony, Hannover, Germany; 6 Department of Health Economics and Health Service Research, University Medical Centre Hamburg-Eppendorf, Hamburg, Germany; 7 Internal Medicine IX – Department of Clinical Pharmacology and Pharmacoepidemiology, Heidelberg University Hospital, Heidelberg, Germany; 8 MHH Information Technology – Science and Laboratory, Hannover Medical School, Hannover, Germany; 9 Institute of General Practice and Family Medicine, Martin-Luther-University Halle-Wittenberg, Halle, Saale, Germany; 10 Institute for Community Medicine, University Medicine Greifswald (UMG), Greifswald, Germany; 11 Faculty V: School of Life Sciences, University of Siegen, Siegen, Germany

**Keywords:** Dementia, Lifestyle intervention, Randomized controlled trial, Behavior change, Transtheoretical model

## Abstract

**Purpose:**

We investigated the effects of a multidomain lifestyle intervention conducted in older adults at increased risk for dementia on participants’ diet.

**Methods:**

Secondary analyses of the cluster-randomized AgeWell.de-trial, testing a multidomain intervention (optimization of nutrition and medication, enhancement of physical, social and cognitive activity) in older adults at increased dementia risk. Intervention effects on a healthy diet (composite score) and its components were analyzed using Poisson- and logistic regression analyses. Stages of behavior change (transtheoretical model), and respective changes between baseline and follow-up were analyzed using mixed regression analyses.

**Results:**

A total of 819 individuals were analyzed (M_age_ = 69.0, SD = 4.9, n_intervention/control group_ = 378/441). We observed a significant intervention effect on the healthy diet score (b = 0.06, IRR: 1.06, 95% CI: 1.01, 1.11). Changes were particularly due to increased fruit- and vegetable consumption, while other food components were not improved by the intervention. The intervention did not induce transitions to advanced stages of behavior change regarding a healthy diet, however, participants in the control group moved to initial stages of behavior change (OR = 1.95, 95% CI: 1.30, 2.92).

**Conclusion:**

A multidomain lifestyle intervention improved participants’ diet and maintained motivation to change in an at-risk-sample. However, only fruit- and vegetable consumption increased. Additional support might be necessary to encourage older adults to integrate new, healthier food components into their diet. Control group participants transitioned to initial stages of behavior change, stressing the need to encourage older adults to maintain a healthy diet as they age.

**AgeWell.de was prospectively registered in the German Clinical Trials Register (DRKS; identifier: DRKS00013555) on December 7th, 2017:**

DRKS00013555

**Supplementary Information:**

The online version contains supplementary material available at 10.1007/s00394-024-03563-z.

## Introduction

Despite recent progress in disease-modifying treatments [1, 2], Alzheimer’s disease and other dementias currently cannot be cured. Due to population ageing, the number of people living with dementia is increasing worldwide, making dementia risk reduction on a scalable level a public health priority [3]. An estimated 1.8 million people in Germany currently live with dementia [4], however, due to low detection rates of mild cognitive impairment in primary care [5, 6], the true number of cases might be even higher. However, knowledge on potentially modifiable risk factors is increasing rapidly, giving rise to the hope for reduction of incident cases through risk factor modification [7].

A healthy diet has been linked to better cardiovascular and brain health. Evidence, though mostly from observational studies, suggests e.g. beneficial effects of the Mediterranean diet or the Dietary Approaches to Stop Hypertension (DASH)-diet on cognitive performance and dementia risk [8–10]. In the Netherlands, the national dietary guidelines were explicitly developed based on studies showing an association of specific dietary components with a reduced risk of chronic diseases, e.g., cardiovascular diseases, diabetes or dementia [11]. It has been shown that the Dutch guidelines are associated with larger brain tissue volumes, indicating better brain health [12].

Data on effectiveness of dietary interventions on cognitive outcomes and brain health is currently scarce. In a recent 3-year randomized controlled trial in older adults with a family history of dementia, an intervention consisting of the MIND-diet with caloric restriction did not improve cognitive performance or MRI-based brain health markers [13]. Changes in cognitive performance might be challenging to observe in lifestyle-based intervention studies, e.g. due to the necessity of extensive follow-up periods [14]. Therefore, it might be promising to assess changes in lifestyle and dementia risk profiles as surrogate outcomes, since respective beneficial changes might reduce dementia risk in the long run [15].

Secondary analyses of the Finnish Geriatric Intervention Study to Prevent Cognitive Impairment and Disability (FINGER [16]) suggest improvements in participants’ diet due to the multi-domain intervention (optimization of nutrition, enhancement of physical, social and cognitive activity) at 24 months follow-up [17]. However, there is still a lack of studies examining in detail how effectively multimodal brain health interventions can promote healthier eating. It has not yet been investigated which aspects of a healthy diet are amendable by lifestyle-based interventions.

Motivation and (health-specific) self-efficacy have been pointed out as potential determinants of a healthy diet [18] and of health behavior change for brain health in (older) adults [19, 20]. The transtheoretical model (TTM) describes successful health behavior change as the passing through different stages of change [21, 22], including: (a) precontemplation, i.e., not engaging in the respective health behavior and no intent to do so; (b) contemplation, where the behavior is not conducted, but considered in the future; (c) preparation, where first small changes are introduced; (d) action, in which behavior change has been introduced but is still perceived as difficult; and (e) maintenance, in which the behavior has been adapted and the person finds it easy to maintain it. In intervention trials, progressing through the stages of change might be an outcome itself, as a more advanced stage increases the probability to change behavior subsequently. Currently, our understanding of the stages of behavior change among older adults at increased risk of dementia is limited, as is our knowledge of the impact of lifestyle-based interventions on these stages.

We therefore investigated:

1. effects of the multidomain AgeWell.de-intervention, conducted in older adults at increased risk for dementia in Germany, on changes in diet, both overall and regarding consumption of specific foods;
2. stages of health behavior change regarding a healthy diet in participants of AgeWell.de and respective changes between baseline and follow-up.

## Methods and materials

### Participants

We report data from the AgeWell.de-trial, a multidomain cluster-randomized controlled lifestyle intervention conducted over 24 months in older adults (60–77 years) at increased risk for dementia, according to a Cardiovascular Risk Factors, Ageing and Dementia (CAIDE [23])-score of ≥ 9 points. Participants were recruited via their general practitioner (GP) at five study sites in Germany (Leipzig, Halle, Munich, Greifswald, Kiel). At baseline (06/2018 – 10/2019) and follow-up, (07/2020 – 01/2022) a total of 1,030 and 819 GP patients participated, respectively. Study design [24], baseline characteristics [25] and trial results [26] are reported in detail elsewhere. Primary outcome was cognitive performance at follow-up; secondary outcomes included (instrumental) activities of daily living, depressive symptoms, social inclusion, and (health-related) quality of life.

### The AgeWell.de multidomain intervention

The intervention group (IG; n_baseline_ = 487) received a multidomain intervention, including advice on optimization of nutrition, enhancement of physical, cognitive, and social activity, optimization of medication and, if applicable, an intervention targeting depressive symptoms and grief. After the baseline examination, conducted as in-person interviews at participants’ homes, study nurses instructed participants on how to conduct the intervention. Optimization of nutrition was based on the national guidelines for a healthy diet [27], which recommend the consumption of a diverse diet, including the consumption of 5 portions of vegetables and fruit a day, limiting consumption of meat and preferring wholegrain over other grain products. The recommendations are very close to the Dutch national guidelines, while the latter are more detailed and have the significant advantage of having been shown to be associated with benefits for brain health [12]. Participants set goals regarding a healthy diet individually with the study nurse after the baseline visit. Control group (CG; n_baseline_ = 543) participants received GP treatment as usual and written information on associations between lifestyle and brain health.

The physical activity-component of the intervention included standardized exercises for strength and balance/flexibility to conduct at home twice/week, and aerobic exercise based on participants’ preferences to be conducted 3–5 times/week. For cognitive activity, participants received tablet PCs with the training software NeuroNation © installed and were told to use the app at least 3 times/week for at least 15 min. Further goals for cognitively demanding activities were defined with participants individually. Participants set individual goals for social activity, based on their preferences. Attending GPs provided information on diagnoses, lab values for hemoglobin A1c and creatinine and prescribed medication. Participants provided information on actual medication. An electronically supported evaluation was conducted based on this information at the AgeWell.de-study site for pharmacology and pharmacoepidemiology at Heidelberg University Hospital, focusing on drugs with high anticholinergic burden, potentially missing medication or potentially serious drug-drug-interactions. If applicable, a letter was sent to the attending GP with suggestions for modification of participants’ medication.

### Outcomes and covariates

We assessed consumption of a healthy diet using a composite score, based on an approach of Voortman and colleagues [28], measuring intake of 11 foods/food groups found to be beneficial for prevention of chronic conditions like cardiovascular disease, diabetes and dementia [28]. One point is given for each of the following items fulfilled, resulting in a total score ranging from 0 to 11:

- Eat ≥ 200 g of vegetables daily
- Eat ≥ 200 g of fruit daily
- Eat ≥ 90 g of wholegrain products daily
- Eat legumes weekly (≥ 135 g/week)
- Eat ≥ 15 g of unsalted nuts daily
- Eat one serving of fish, preferably oily fish, weekly (≥ 100 g/week)
- Drink three cups of tea daily (≥ 450 ml/day)
- Take a few portions of dairy produce daily (≥ 350 g/day)
- Limit the consumption of red meat, particularly processed meat (< 300 g/week)
- Minimize the consumption of sugar-containing beverages (< 150 ml/day)
- Do not drink alcohol or no more than one glass daily (< 10 g/day).

Participants’ diet was assessed at baseline and follow-up using a validated food frequency questionnaire (FFQ; [29]). The FFQ assessed the consumption frequency over the past month and the number of portions consumed for 53 food items, using the following frequency categories: never; once a month; two to three times a month; one to two times a week; three to four times a week; five to six times a week; one time per day; two times per day; three times per day; four to five times per day, and more than five times per day. Based on consumption frequency and portion sizes, the consumption in grams or milliliters was calculated. Substituting the main analysis using the composite score for a healthy diet, we assessed intervention effects on the consumption of the single food items.

As covariates, we included information on sex, age, education (low/middle/high, assessed using the Comparative Analysis of Social Mobility in Industrial Nations (CASMIN)-scale [30]), and marital status (married or cohabitating vs. single, divorced, widowed), assessed at the baseline examination, respectively. To account for potential baseline imbalances in consumption of a healthy diet between groups and to counteract possible regression to the mean [31, 32], we further controlled for baseline values of the healthy diet score and the single food items, respectively.

We assessed participants’ stage of health behavior change exemplarily for fruit and vegetable consumption, with response options operationalized in relation to the TTM [21, 22]. Participants answered the question: “Do you eat at least five portions of fruit and vegetables daily?”, response options being: “no, and I do not intend to” (precontemplation stage); “no, but I am thinking about it” (contemplation stage), “no, but I intend to do so” (preparation stage); “yes, but I find it hard” (action stage); “yes, and I find it easy” (maintenance stage).

### Statistical analyses

Descriptive statistics are presented for the overall sample and separately for IG and CG. In multivariable analyses, an intervention effect on a healthy diet was examined. A healthy diet was operationalized as a count variable over eleven components of a healthy diet, therefore, a Poisson regression was calculated. Clustering of participants in GP practices was accounted for by cluster-robust standard errors. Logistic regressions were performed to investigate which of the eleven diet components were affected by the intervention.

Inspection of incomplete data revealed no evidence of systematically missing values; missing values were assumed to be missing at random (MAR) and imputed using multiple imputation by chained equations, with regression analyses based on pooled results of 50 imputed datasets. The imputations were performed at the item level of the FFQ, and the diet score was subsequently calculated. The p-value was set at 0.05 for all analyses (two-tailed). In the logistic regressions, examining the intervention effect on eleven diet components, a Bonferroni correction for multiple testing was applied (*p* < 0.005).

To investigate whether the distribution of stages of health behavior change according to the transtheoretical model differed between IG and CG, we calculated mixed models for ordinal data with the between-subjects factor group membership and the within-subjects factor time point (baseline vs. follow-up). All analyses were conducted using Stata 16.1 (StataCorp).

## Results

### Descriptive analyses

Table 1 describes baseline characteristics of the analyzed sample. Participants in the IG and CG differed slightly in levels of education (*p* = 0.032), with more IG-participants having a low level of education. More CG- than IG-participants consumed the recommended amount of tea at baseline (*p* = 0.006). No further differences between groups were detected at baseline. The number of missing values at baseline ranged from 1.8% (consumption of nuts) to 14.3% (alcohol consumption). At follow-up, the number of missing values ranged from 13.6% (consumption of nuts, consumption of legumes) to 23.1% (alcohol consumption). Participants who were married or cohabitating were more likely to have missing values for consumption of red/processed meat (*p* = 0.017), alcohol (*p* = 0.006) and vegetables (*p* < 0.001) at follow-up. Likelihood of missing values for any item of the FFQ was not linked to age, sex, education, BMI or a diagnosis of diabetes.

**Table 1:** Description of participant characteristics at baseline

	n	Total sample (n = 819)	Intervention (n = 378)	Control (n = 441)	*p*
Sociodemographic and health-related factors					
Age, *M (SD)*	819	69.0 (4.9)	69.0 (4.9)	69.0 (4.9)	.769^a^
Female sex, *n (%)*	819	433 (52.9)	199 (52.7)	234 (53.1)	.905^b^
Married/cohabitating, *n (%)*	819	532 (65.0)	247 (65.3)	285 (64.6)	.830^b^
Education, *n (%)*	819				.032*^b^
Low		181 (22.1)	98 (25.9)	83 (18.8)	
Medium		434 (53.0)	196 (51.9)	238 (54.0)	
High		204 (24.9)	84 (22.2)	120 (27.2)	
Diabetes, *n (%)*	814	311 (38.2)	136 (36.1)	175 (40.1)	.245^b^
Body mass index, *M (SD)*	810	31.0 (5.4)	31.0 (5.3)	30.9 (5.6)	.777^a^
Healthy diet					
Diet score, *M (SD)*	519	4.2 (1.6)	4.2 (1.6)	4.2 (1.7)	.929^a^
Vegetables ≥ 200 g/day, *n (%)*	749	146 (19.5)	62 (17.9)	84 (20.9)	.297^b^
Fruit ≥ 200 g/day, *n (%)*	783	370 (47.3)	179 (48.8)	191 (45.9)	.424^b^
Whole grains ≥ 90 g/day, *n (%)*	790	293 (37.1)	135 (36.4)	158 (37.3)	.701^b^
Legumes ≥ 135 g/week, *n (%)*	795	161 (20.3)	74 (20.1)	87 (20.4)	.926^b^
Nuts ≥ 15 g/day, *n (%)*	801	92 (11.5)	40 (10.6)	52 (12.2)	.479^b^
Fish ≥ 100 g/week, *n (%)*	756	428 (56.6)	195 (54.9)	233 (58.1)	.379^b^
Tea ≥ 450 ml/day, *n (%)*	778	48 (6.2)	13 (3.6)	35 (8.4)	.006*^,b^
Dairy ≥ 350 g/day, *n (%)*	754	246 (32.6)	113 (32.4)	133 (32.8)	.893
Red and processed meat < 300 g/week, *n (%)*	704	233 (33.1)	119 (35.5)	114 (30.9)	.192^b^
Sugar-containing beverages < 150 ml/day, *n (%)*	776	711 (91.6)	329 (91.6)	382 (91.6)	.985^b^
Alcohol < 10 g/day, *n (%)*	702	441 (62.8)	212 (65.8)	229 (60.3)	.128^b^

*M* mean, *SD* standard deviation

^a^t-test

^b^χ2-test

*p < 0.05

### Intervention effects on diet

Effects of the intervention on a healthy diet (total score) are presented in Table 2. The intervention improved participants’ diet (b = 0.06, IRR: 1.06, 95% CI: 1.01, 1.11). Higher values of the healthy diet score at baseline (b = 0.12, IRR: 1.13, 95% CI. 1.11, 1.15), higher age (b = 0.01, IRR: 1.01, 95% CI: 1.00, 1.01) and female sex (b = 0.08, IRR: 1.08, 95% CI: 1.03, 1.13) were further linked to a healthier diet at follow-up. No effects were detected for level of education or being married/cohabitating.

**Table 2:** Results of the Poisson regression to investigate the intervention effect on a healthy diet at follow-up (total score)

	b	SE	IRR	IRR 95% CI		*p*	
				Lower	Upper		
Baseline diet score	0.12	0.01	1.13	1.11	1.15	< .001	***
Intervention group (ref.: control group)	0.06	0.02	1.06	1.01	1.11	.015	*
Sociodemographic factors							
Age	0.01	0.00	1.01	1.00	1.01	.043	*
Female sex	0.08	0.02	1.08	1.03	1.13	.001	**
Married/cohabitating	0.01	0.03	1.01	0.95	1.07	.778	
Education level medium (ref.: low)	− 0.05	0.03	0.95	0.90	1.01	.100	
Education level high	− 0.05	0.03	0.95	0.89	1.01	.122	

Regression analyses based on pooled results of 50 imputed datasets

*b* coefficient, *SE* standard error, *IRR* incidence rate ratio, *CI* confidence interval, *ref.* reference

*p < .05

**p < .01

***p < .001

Next, we assessed intervention effects on individual components of the healthy diet score (Tables 3, 4). The intervention improved consumption of vegetables (OR = 1.52, 95% CI: 1.03, 2.24) and fruit (OR = 1.87, 95% CI: 1.35, 2.58). For all components of the healthy diet score, higher values at baseline were linked to higher odds of meeting the respective recommendations at follow-up. Higher age was linked to higher odds of consuming the recommended amounts of whole-grain products (OR: 1.05, 95% CI: 1.01, 1.09) and legumes (OR: 1.04, 95% CI: 1.00, 1.08) at follow-up. Women had higher odds of consuming the recommended amounts of vegetables (OR: 1.85, 95% CI: 1.22, 2.80) and fruit (OR: 1.80, 95% CI: 1.32, 2.46). Female sex was further linked to limited consumption of red/processed meat (OR: 2.02, 95% CI: 1.35, 3.03), sugar-containing beverages (OR: 2.34, 95% CI: 1.15, 4.76) and alcohol (OR: 2.12, 95% CI: 1.37, 3.27), however, women less often met the guidelines for consumption of legumes (OR: 0.68, 95% CI: 0.47, 0.97). Medium (OR: 0.57, 95% CI: 0.37, 0.88) and high (OR: 0.54, 95% CI: 0.33, 0.88) levels of education were linked to lower odds of eating the recommended amount of legumes at follow-up, while a high level of education was associated with lower odds of low/moderate alcohol-consumption (OR: 0.50, 95% CI: 0.29, 0.85).

**Table 3:** Results of the logistic regressions to investigate the intervention effect on diet score components

	OR (95% CI) for vegetables ≥ 200 g/day	OR (95% CI) for fruit ≥ 200 g/day	OR (95% CI) for whole grain products ≥ 90 g/day	OR (95% CI) for legumes ≥ 135 g/week	OR (95% CI) for nuts ≥ 15 g/day	OR (95% CI) for fish ≥ 100 g/week
Baseline consumption	4.29 (2.81, 6.54)***^†^	4.74 (3.51, 6.40)***^†^	3.87 (2.83, 5.29)***^†^	4.43 (3.01, 6.53)***^†^	14.66 (8.87, 24.24)***^†^	6.36 (4.44, 9.12)***^†^
Intervention group (ref.: control group)	1.52 (1.03, 2.24)*	1.87 (1.35, 2.58)***^†^	0.96 (0.67, 1.37)	0.99 (0.69, 1.41)	1.29 (0.80, 2.07)	1.13 (0.79, 1.60)
Sociodemographic factors						
Age	0.99 (0.95, 1.03)	1.00 (0.96, 1.03)	1.05 (1.01, 1.09)**	1.04 (1.00, 1.08)*	0.99 (0.94, 1.04)	1.00 (0.97, 1.03)
Female sex	1.85 (1.22, 2.80)**^†^	1.80 (1.32, 2.46)***^†^	0.96 (0.69, 1.32)	0.68 (0.47, 0.97)*	1.16 (0.76, 1.79)	1.09 (0.74, 1.61)
Married/cohabitating	1.12 (0.73, 1.71)	0.86 (0.59, 1.27)	1.15 (0.79, 1.67)	1.16 (0.74, 1.83)	0.95 (0.57, 1.59)	1.03 (0.69, 1.54)
Education level medium (ref.: low)	0.64 (0.38, 1.07)	0.89 (0.59, 1.36)	1.33 (0.91, 1.95)	0.57 (0.37, 0.88)*	1.20 (0.60, 2.40)	0.85 (0.54, 1.32)
Education level high	0.68 (0.39, 1.17)	1.36 (0.84, 2.19)	0.97 (0.62, 1.51)	0.54 (0.33, 0.88)*	0.98 (0.45, 2.14)	0.90 (0.53, 1.52)

Outcome: Odds of consuming the recommended amount of the respective components of a healthy diet (yes/no) at follow-up

Regression analyses based on pooled results of 50 imputed datasets

*CI* confidence interval, *OR* odds ratio

*p < .05

**p < .01

***p < .001 (unadjusted p-values)

†p < .005 (Bonferroni-adjusted p-value)

**Table 4:** Results of the logistic regressions to investigate the intervention effect on diet score components

	OR (95% CI) for tea ≥ 450 ml/day	OR (95% CI) for dairy ≥ 350 g/day	OR (95% CI) for red and processed meat < 300 g/week	OR (95% CI) for sugar-containing beverages < 150 ml/day	OR (95% CI) for alcohol < 10 g/day
Baseline consumption	77.45 (28.01, 214.16)***^†^	7.09 (4.92, 10.22)***^†^	5.20 (3.49, 7.76)***^†^	9.16 (4.57, 18.33)***^†^	8.85 (5.75, 13.64)***^†^
Intervention group (ref.: control group)	0.52 (0.22, 1.26)	0.82 (0.55, 1.21)	1.36 (0.91, 2.03)	1.37 (0.73, 2.55)	0.90 (0.61, 1.33)
Sociodemographic factors					
Age	1.05 (0.96, 1.13)	1.02 (0.99, 1.06)	1.02 (0.98, 1.06)	1.01 (0.94, 1.09)	1.02 (0.98, 1.07)
Female sex	1.57 (0.58, 4.24)	0.91 (0.63, 1.31)	2.02 (1.35, 3.03)**^†^	2.34 (1.15, 4.76)*	2.12 (1.37, 3.27)**^†^
Married/cohabitating	0.63 (0.25, 1.58)	1.11 (0.75, 1.64)	1.05 (0.70, 1.57)	0.59 (0.26, 1.35)	1.04 (0.65, 1.66)
Education level medium (ref.: low)	0.97 (0.31, 2.99)	0.82 (0.51, 1.33)	1.06 (0.64, 1.73)	0.74 (0.32, 1.71)	0.73 (0.45, 1.20)
Education level high	0.85 (0.23, 3.19)	0.94 (0.57, 1.57)	1.35 (0.81, 2.25)	1.35 (0.49, 3.71)	0.50 (0.29, 0.85)*

Outcome: Odds of consuming the recommended amount of the respective components of a healthy diet (yes/no) at follow-up

Regression analyses based on pooled results of 50 imputed datasets

*CI* confidence interval, *OR* odds ratio

*p < .05

**p < .01

***p < .001

†p < .005 (Bonferroni-adjusted p-value)

Changes in the healthy diet score and proportions of IG- and CG-participants consuming the recommended amounts of the single respective foods from baseline to follow-up, assessed using Poisson- and logistic regression, are described in Table 5. The improvement in the healthy diet score from baseline to follow-up was greater in the IG compared to the CG (*p* = 0.015). While fruit- and vegetable-intake increased in the IG, the share of CG-participants consuming the recommended amounts of fruit and vegetables decreased from baseline to follow-up (*p* = 0.041, *p* < 0.001, respectively). Differences in change between groups were not significant for the other foods.

**Table 5:** Changes in the diet score and diet score components from baseline to follow-up

	Total sample (n = 819)	Intervention (n = 378)	Control (n = 441)	*p*^a^
Healthy diet				
∆ Diet score (mean)	+ 0.2	+ 0.4	+ 0.1	.015*
Healthy diet components ^a^				
∆ Vegetables ≥ 200 g/day in %	+ 1.8	+ 6.9	− 3	.041*
∆ Fruit ≥ 200 g/day in %	− 1	+ 5.1	− 6.7	< .001***^†^
∆ Whole grains ≥ 90 g/day in %	+ 1.8	+ 1.7	+ 1.9	.796
∆ Legumes ≥ 135 g/week in %	+ 6.3	+ 6.9	+ 5.8	.926
∆ Nuts ≥ 15 g/day in %	+ 3.3	+ 5.1	+ 1.8	.296
∆ Fish ≥ 100 g/week in %	+ 2.4	+ 5.0	0	.484
∆ Tea ≥ 450 ml/day in %	− 1.2	− 0.8	− 1.3	.195
∆ Dairy ≥ 350 g/day in %	− 3.7	− 5.1	− 2.4	.290
∆ Red and processed meat < 300 g/week in %	+ 3.9	+ 5.7	+ 1.9	.138
∆ Sugar-containing beverages < 150 ml/day in %	+ 1.8	+ 2.7	+ 1	.290
∆ Alcohol < 10 g/day in %	+ 2.9	+ 0.8	+ 4.7	.709

Regression analyses based on pooled results of 50 imputed datasets

*p < .05

**p < .01

***p < .001 (unadjusted p-values)

†p < .005 (Bonferroni-adjusted p-value)

^a^Results of Poisson regression (total score) and logistic regressions (healthy diet components) adjusted for baseline diet, age, sex, marital status and education

Complementing the analyses of intervention effects on a healthy diet (total score) and its respective components based on imputed datasets, we conducted sensitivity analyses using unimputed data, revealing highly comparable results (Supplementary Material 1).

### Changes in stages of health behavior change

Table 6 describes distributions of participants across the stages of health behavior change regarding a healthy diet at baseline and follow-up. At baseline, there were no significant differences between IG and CG (*p* = 0.940 and *p* = 0.660). However, at follow-up, significantly more people in the CG had moved to the precontemplation stage (*p* < 0.001).

**Table 6:** Distribution of participants across the different stages of health behavior change according to the transtheoretical model

Do you eat at least five portions of fruit and vegetables every day?		Stage of health behavior change	Number (%) of participants		
Baseline					
			Intervention	Control	*p*
*No, and…*					.940
	*I do not intend to*	Precontemplation	81 (21.7)	97 (22.2)	
	*I think about it*	Contemplation	80 (21.4)	103 (23.6)	
	*I firmly intend to*	Preparation	26 (7.0)	33 (7.6)	
*Yes, and…*					.660
	*I find it difficult*	Action	20 (5.3)	19 (4.4)	
	*I find it easy*	Maintenance	167 (44.7)	184 (42.2)	
			Σ 374	Σ 436	
Follow-up					
			Intervention	Control	*p*
*No, and…*					< .001
	*I do not intend to*	Precontemplation	75 (21.7)	166 (40.8)	
	*I think about it*	Contemplation	67 (19.4)	52 (12.8)	
	*I firmly intend to*	Preparation	15 (4.3)	14 (3.4)	
*Yes, and…*					.200
	*I find it difficult*	Action	29 (8.4)	19 (4.7)	
	*I find it easy*	Maintenance	159 (46.1)	156 (38.3)	
			Σ 345	Σ 407	

**Fig. 1:**
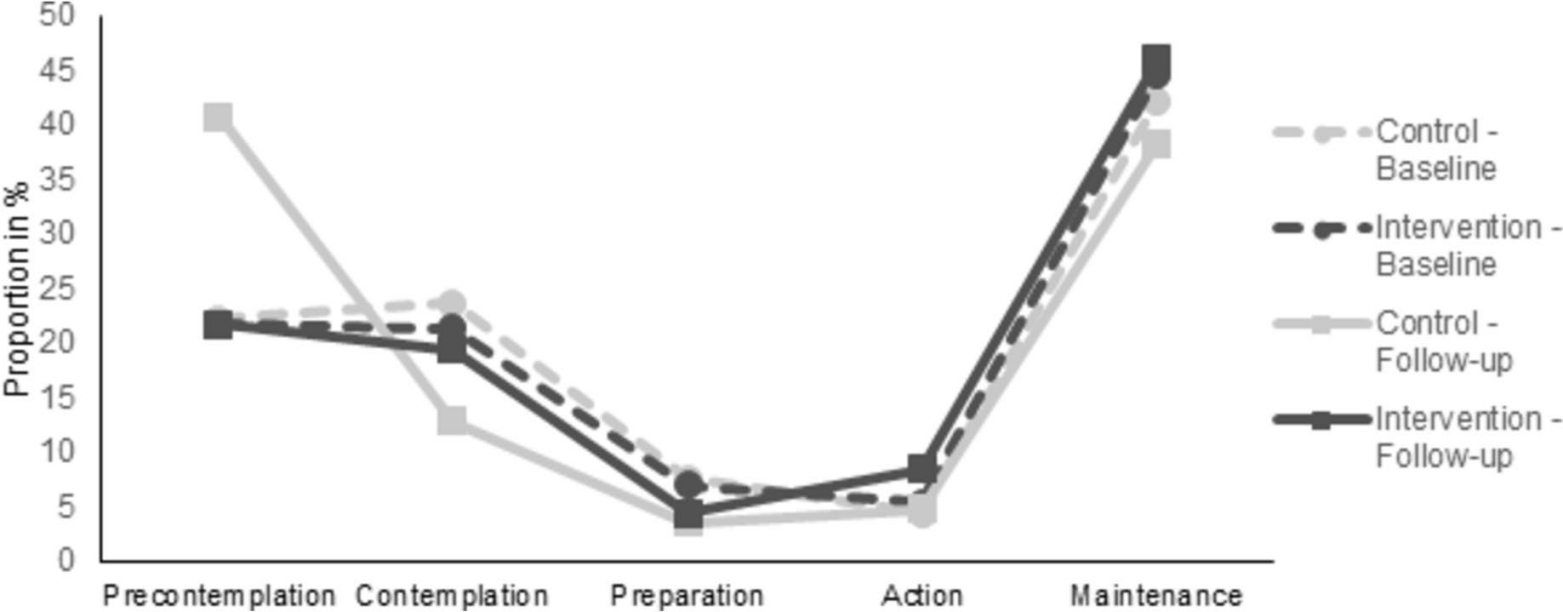
Distribution of intervention- and control group participants across stages of health behavior change at baseline and follow-up

In the mixed regression analysis for ordinal data, there was no significant difference between IG and CG regarding stages of health behavior change (*p* = 0.494; not tabulated). We observed a significant main effect for time, with participants moving to initial stages of health behavior change at follow-up (OR = 0.54, 95% CI: 0.41, 0.71, *p* < 0.001). There was a significant group*time-interaction, indicating that only CG-participants moved to initial stages of change, which was not the case for the IG (OR = 1.95, 95% CI: 1.30, 2.92, *p* = 0.001; Fig. 1). Supplementing these analyses, we calculated a logistic regression, analyzing changes from the precontemplation-, contemplation- or preparation-stage (0) to the action- or maintenance-stage (1; not tabulated). Results revealed a significant group*time-interaction, with CG-participants being less likely to transition to the action- or maintenance-stage at follow-up (OR = 0.64, 95% CI: 0.42, 0.92, *p* = 0.018).

Since dietary recommendations, e.g., by the attending GP, might slightly differ for persons with diabetes or obesity, we conducted sensitivity analyses for intervention effects on a healthy diet (total score, components), changes in the diet score and its components and stages of health behavior change, including body mass index (weight [kg]/height[m]^2^) and diabetes (diagnosed by GP) as covariates. Results for the respective outcomes did not change when including BMI and diabetes into regression analyses (not tabulated).

## Discussion

This study assessed effects of a 2-year multidomain lifestyle intervention on a healthy diet in older adults at increased risk for dementia. Further, we investigated changes in stages of health behavior change regarding a healthy diet between baseline and follow-up. Using data from the AgeWell.de-study, we found beneficial intervention effects on participants’ diet at follow-up. We detected improvements in a composite healthy diet score and in intake of the recommended amounts of fruit and vegetables, respectively. The intervention did not induce transitions to advanced stages of behavior change regarding a healthy diet, however, CG participants moved to initial stages of behavior change during the intervention period.

Our findings are in line with a study by Lehtisalo and colleagues, reporting a beneficial effect of the FINGER-intervention on a composite score of a healthy diet [17]. The multidomain Healthy Ageing Through Internet Counseling in the Elderly (HATICE)-trial did not find intervention effects on adherence to a Mediterranean diet ([33]). These studies are, to the best of our knowledge, the only completed multidomain intervention trials targeting older adults at increased risk for dementia/cardiovascular disease describing intervention effects on nutrition. The PREMIER-study [34], applying a multicomponent intervention in adults with hypertension, increased intake of fruit, vegetables, fiber and minerals and decreased intake of sodium. While this intervention included adults aged 25 and older, our findings suggest that multidomain lifestyle-based interventions can provide positive effects on diet similarly in older age groups. Other single-domain intervention trials have reported beneficial effects of counseling/health education and self-management on a healthy diet [35, 36] in older adults, which is comparable to the nutritional intervention component applied in AgeWell.de.

The observed changes in the healthy diet score were particularly due to increased intake of fruit and vegetables, while several other foods comprised by the healthy diet score (e.g., nuts, legumes, dairy, fish) were not amendable by the intervention. Comparable results have been reported for FINGER, where no changes in the consumption of nuts, legumes or sweetened beverages were observed [17]. Consumption of fruit and vegetables is recommended unequivocally in (national) guidelines for a healthy diet and has been advertised in public health campaigns for several decades. This may imply an overall higher awareness of their role for a healthy diet than of other foods. In line with this, awareness regarding dietary guidelines was highest for regular consumption of fruit and vegetables in a representative survey in German adults [37]. More effort might be needed to raise awareness for and increase openness towards consumption of other healthy foods. What is more, several foods included in the applied healthy diet score are usually not consumed on a daily, but rather on a weekly basis, e.g., fish or legumes. Our findings suggest that interventions targeting nutrition should not only aim at increasing the daily intake of certain foods, but also at introducing new ingredients into participants’ diet. Similar patterns were found in a qualitative study on lifestyle behaviors for brain health from the Netherlands, were participants (adults aged 40–79 years) favored intensifying behaviors beneficial for brain health they were already engaging in, but were less inclined to integrate new habits into their lifestyle [38]. Providing participants with recipes and suggestions on how to combine certain foods or setting specific goals encouraging the intake of “new” foods might constitute promising approaches for future trials. Food diaries to track consumption of foods might further aid in monitoring participants’ adherence to a healthy and diverse diet.

Taking a closer look at changes in consumption of single foods, we found evidence for decreased intake of fruit and vegetables in the CG in the course of the trial. Although no respective changes were observed for the other foods/food groups, this finding is noteworthy, since dietary quality becomes ever more important with decreasing overall energy intake typically observed with ageing [39, 40]. This highlights the importance of supporting older adults in adopting or maintaining a healthy diet to age healthily. On another note, our findings might have been affected by the COVID-19 pandemic, which afflicted large parts of the intervention period: While measures to curb spreading of the virus, e.g., lockdowns, shutdowns of local facilities etc. affected all participants, CG-participants did not receive additional support regarding maintenance/uptake of a healthy diet. It might be that the intervention provided additional support for IG-participants, which enabled them to maintain or improve their dietary habits, leading to the observed between-group differences in change of fruit/vegetable consumption at follow-up. Absence of motivation and support within the CG during the pandemic might also partially explain transitions to initial stages of motivation for behavior change. This would be in line with findings from the Netherlands, reporting unhealthy changes in older adults’ diet during the pandemic [41], while other studies did not observe negative impacts of the pandemic on older adults’ dietary behavior [42, 43]. As reported earlier [26], IG-participants more often reported perceived restrictions regarding adherence to the nutrition-component of the multidomain intervention. Since, however, we did not assess specific changes in participants’ diet during the pandemic, and other explanations for the observed effects cannot be ruled out, this finding should be interpreted cautiously.

Regarding the stages of behavior change according to the TTM, the intervention did not induce transitions to more advanced stages of health behavior change; rather, we detected transitions to initial stages of change in the CG. This might, at least in part, be explained by the study design: Since all participants agreed to participate in a lifestyle intervention targeting, amongst others, a healthy diet, a high baseline-level of motivation for behavior change could be expected. Indeed, almost half of participants both in IG and CG were in the action- or maintenance-stage regarding fruit and vegetable consumption at baseline. Similar findings were reported by Clark and colleagues, with high proportions of participants in the action- and maintenance-stage at the baseline-examination of the SENIOR-trial [44]. Population-based samples, on the other hand, generally report more even distributions of individuals across the stages of change (e.g., [45]). Probably, the AgeWell.de-intervention was able to maintain participants’ motivation throughout the intervention period, while participants in the CG transitioned to initial stages of behavior change. However, our findings regarding the stages of behavior change are consistent with the reported decreased consumption of fruits and vegetables in the CG.

AgeWell.de followed a pragmatic trial design with the aim of practicable implementation in real-world settings, therefore, the intervention (including the component on healthy diet) was conducted independently by participants. More guided activities like e.g. cooking classes and interaction with other participants for peer support might increase motivation and self-efficacy for behavior change, which may favor transition to advanced stages of behavior change and, possibly, increase participants’ likelihood to integrate new foods into their diet. Including specific intervention components to strengthen motivation and self-efficacy, supporting participants in setting SMART (specific, measurable, achievable, relevant, time-limited) goals and providing strategies to deal with relapses to old behaviors might increase intervention effectiveness in future trials [46, 47].

### Strengths and limitations

Strengths of our study include the randomized trial design, allowing for statements on effectiveness of a lifestyle-based intervention on older adults’ diet. While the number of (multidomain) lifestyle-based interventions targeting healthy ageing is steadily increasing, knowledge on the effects of corresponding interventions on older adults’ diet is still scarce. While much of our knowledge on older adults’ nutrition is focusing on intake of fruit and vegetables, we were able to assess a variety of foods important for healthy ageing. We used a composite healthy diet score based on national nutritional guidelines, which has already been associated with better brain health [12]. This might be a more promising measure of dietary quality, as research on nutrition and ageing has shifted from identifying a magic bullet and examining the roles of foods/nutrients in isolation to a focus on the effects of combinations/patterns of foods and nutrients on healthy ageing [48, 49].

Several limitations should be noted when interpreting our findings. The IG conducted the intervention independently and set personal goals for a healthy diet and further intervention components, following an instruction by the study nurse. Therefore, intensity of the intervention may have varied between participants. This, however, might also imply that multidomain interventions which are less intensive than the original FINGER-intervention and are conducted independently can improve participants’ diet. Older adults at increased risk for dementia, participating in a multidomain lifestyle intervention, constitute a slightly selective sample. This may have influenced our findings, especially regarding the stages of behavior change, with high proportions of participants in both groups in advanced stages of change at baseline, possibly limiting generalizability of respective findings to population-based samples. Since the CG did not receive an intervention, no between-group comparison regarding adherence to the intervention and respective effects on between-group differences at follow-up regarding a healthy diet were feasible. Self-reporting of food intake raises the risk of bias, e.g., due to social desirability. However, moderate overall scores for a healthy diet and diet score components argue against a general tendency for overestimation in our study. We assessed stages of health behavior change according to the transtheoretical model asking specifically about fruit and vegetable consumption, which precludes us from making statements about changes in stages of health behavior change for the other components of the healthy diet score. However, due to the important role of fruit and vegetable consumption for a healthy diet, our results should still be interpretable as meaningful. Due to budget limitations, we were not able to collect biomarkers which could have given a more objective picture of changes in nutrition, including health benefits like e.g. vitamin intake. To keep burden for participants low, we assessed food intake at baseline and follow-up, without additional assessments throughout the trial. Respective interim-assessments of participants’ diet may have provided a more nuanced picture of changes in diet over time. Lastly, our data did not allow for a feasible assessment of factors like sodium intake, which has been linked to healthy ageing and cognitive decline, due to the nature of the FFQ applied in AgeWell.de. Additional assessments, e.g., urinary measurements or alternative FFQs may provide useful information in future trials.

## Conclusion

A pragmatic lifestyle-based multidomain intervention improved participants’ diet after a two-year intervention period and maintained motivation to change diet behavior. These beneficial changes might contribute to reduced risks for several chronic diseases, if maintained beyond the intervention period. Results suggest that a healthy diet might constitute a useful surrogate outcome for future intervention studies targeting healthy ageing. However, only certain food groups, i.e., fruits and vegetables, were amendable by the intervention. More tailored interventions and support of participants might be required to initiate or increase consumption of other foods as well and increase intervention effectiveness. Participants in the control group transitioned to initial stages of behavior change regarding a healthy diet. Considering the health risks of malnutrition in older age, and the potential benefits of a diverse and healthy diet, this finding points towards the need for supporting older adults in adopting and maintaining a healthy diet.

## Supplementary Information

Below is the link to the electronic supplementary material.

Supplementary file1 (PDF 227 KB)

## Data Availability

The data underlying the current study are not publicly available due to privacy restrictions. Data are available after de-identification to researchers who submit a sound proposal to the AgeWell.de steering committee. Respective requests should be submitted to: Steffi.Riedel-Heller@medizin.uni-leipzig.de.
