# Supplementary material for: Dietary changes following a lifestyle-based intervention for dementia risk reduction – results from the AgeWell.de study
Source: Eur J Nutr. 2024 Dec 30;64(1):58. doi: 10.1007/s00394-024-03563-z (PMC11685242; doi:10.1007/s00394-024-03563-z)
Supplement: Supplementary file 1 — Supplementary file1 (PDF 227 KB) [file 394_2024_3563_MOESM1_ESM.pdf]

Supplementary Material 1

Zuelke, Blotenberg et al., Dietary changes following a lifestyle-based intervention for dementia risk reduction – Results from the AgeWell.de study

eTable 1. Results of the Poisson regression to investigate the intervention effect on a healthy diet based on unimputed data (total score).

	b	SE	IRR	IRR 95% CI		*p*	
				Lower	Upper		
Baseline diet score	0.13	0.01	1.14	1.12	1.16	< .001	***
Intervention group	0.08	0.03	1.08	1.02	1.16	.015	*
Sociodemographic factors							
Age	0.00	0.00	1.00	1.00	1.01	.253	
Female sex	0.03	0.04	1.03	0.96	1.10	.437	
Married / cohabitating	0.02	0.04	1.02	0.93	1.11	.697	
Education low (reference)							
Education level medium	-0.07	0.04	0.93	0.86	1.01	.092	
Education level high	-0.14	0.05	0.87	0.79	0.96	.006	**

Notes. SE = Standard Error, IRR = Incidence Rate Ratio, CI = Confidence Interval

eTable 2a. Results of the logistic regressions to investigate the intervention effect on diet score components based on unimputed data.

	OR for vegetables ≥ 200 g/day	OR for fruit ≥ 200 g/day	OR for whole grain products ≥ 90 g/day	OR for legumes ≥ 135 g/week	OR for nuts ≥ 15 g/day	OR for fish ≥ 100 g/week
Baseline consumption	4.83 (3.11, 7.52) ***†	5.24 (3.79, 7.24)***†	4.41 (3.18, 6.12)***†	5.13 (3.47, 7.58)***†	14.93 (9.18, 24.28)***†	10.09 (6.70, 15.20)***†
Intervention group	1.64 (1.10, 2.45)*	2.01 (1.42, 2.83)***†	0.97 (0.67, 1.40)	1.04 (0.71, 1.51)	1.29 (0.81, 2.06)	1.19 (0.83, 1.71)
Sociodemographic factors						
Age	0.98 (0.94, 1.02)	1.00 (0.96, 1.03)	1.05 (1.01, 1.09)**	1.04 (1.00, 1.08)*	0.99 (0.94, 1.04)	1.00 (0.97, 1.03)
Female sex	1.86 (1.22, 2.85)**†	1.79 (1.29, 2.48)***†	0.94 (0.68, 1.30	0.68 (0.47, 0.98)*	1.16 (0.77, 1.76)	1.09 (0.73, 1.63)
Married / cohabitating	1.18 (0.76, 1.82)	0.84 (0.57, 1.26)	1.17 (0.79, 1.73)	1.09 (0.68, 1.74)	0.93 (0.56, 1.56)	1.09 (0.71, 1.67)
Education low (reference)						
Education level medium	0.60 (0.35, 1.02)	0.93 (0.62, 1.41)	1.45 (0.98, 2.15)	0.54 (0.34, 0.88)*	1.24 (0.62, 2.49)	0.95 (0.59, 1.51)
Education level high	0.54 (0.31, 0.92)	1.35 (0.83, 2.19)	0.98 (0.63, 1.52)	0.52 (0.31, 0.87)*	0.98 (0.45, 2.13)	0.91 (0.51, 1.61)

Notes. OR = Odds Ratio. * p < .05, ** p < .01, *** p < .001 (unadjusted p-values). † p < .005 (Bonferroni-adjusted p-value). Outcome: Odds of consuming the recommended amount of the respective components of a healthy diet (yes/no) at follow-up

eTable 2b. Results of the logistic regressions to investigate the intervention effect on diet score components based on unimputed data.

	OR for tea ≥ 450 ml/day	OR for dairy ≥ 350 g/day	OR for red and processed meat < 300 g/week	OR for sugar-containing beverages < 150 ml/day	OR for alcohol < 10 g/day
Baseline consumption	112.91 (37.99, 335.59)***†	9.46 (6.56, 13.62)***†	7.78 (4.83, 12.55)***†	13.49 (6.32, 28.78)***†	12.03 (7.76, 18.64)***†
Intervention group	0.45 (0.19, 1.09)	0.81 (0.52, 1.27)	1.46 (0.90, 2.36)	1.48 (0.75, 2.91)	0.91 (0.60, 1.37)
Sociodemographic factors					
Age	1.05 (0.97, 1.15)	1.03 (1.00, 1.07)	1.02 (0.98, 1.07)	1.02 (0.93, 1.11)	1.04 (0.99, 1.09)
Female sex	2.10 (0.74, 5.94)	0.79 (0.53, 1.16)	2.56 (1.63, 4.04)***†	2.88 (1.39, 5.94)**	2.23 (1.36, 3.64)**†
Married / cohabitating	0.61 (0.24, 1.50)	1.06 (0.69, 1.64)	1.29 (0.80, 2.06)	0.41 (0.17, 1.03)	1.07 (0.63, 1.82)
Education low (reference)					
Education level medium	0.86 (0.28, 2.59)	0.66 (0.38, 1.15)	0.81 (0.45, 1.46)	0.76 (0.33, 1.72)	0.72 (0.42, 1.26)
Education level high	0.70 (0.19, 2.53)	0.94 (0.52, 1.69)	1.20 (0.67, 2.15)	1.60 (0.57, 4.45)	0.48 (0.27, 0.84)*

Notes. OR = Odds Ratio. Unadjusted p-values: * $p < .05$, ** $p < .01$, *** $p < .001$. Bonferroni-adjusted p-value: † $p < .005$. Outcome: Odds of consuming the recommended amount of the respective components of a healthy diet (yes/no) at follow-up
